# Effect of Kenaf Fibre as Reinforcing Fillers in Corn Starch-Based Biocomposite Film

**DOI:** 10.3390/polym14081590

**Published:** 2022-04-13

**Authors:** M. D. Hazrol, S. M. Sapuan, E. S. Zainudin, N. I. A. Wahab, R. A. Ilyas

**Affiliations:** 1 Advanced Engineering Materials and Composites Research Centre, Department of Mechanical and Manufacturing Engineering, Universiti Putra Malaysia (UPM), Serdang 43400, Malaysia; hazrolpostgrad@gmail.com (M.D.H.); sapuan@upm.edu.my (S.M.S.); 2 Laboratory of Biocomposite Technology, Institute of Tropical Forestry and Forest Products (INTROP), Universiti Putra Malaysia (UPM), Serdang 43400, Malaysia; ahmadilyas@utm.my; 3 Department of Electrical and Electronic Engineering, Universiti Putra Malaysia (UPM), Serdang 43400, Malaysia; izzri@upm.edu.my; 4 School of Chemical and Energy Engineering, Faculty of Engineering, Universiti Teknologi Malaysia, Johor Bahru 81310, Malaysia; 5 Centre for Advanced Composite Materials (CACM), Faculty of Engineering, Universiti Teknologi Malaysia, Johor Bahru 81310, Malaysia

**Keywords:** corn starch, kenaf fibre, cellulose, physical properties, tensile properties, water barrier properties

## Abstract

Biocomposite films were prepared using corn starch (CS), sorbitol as a plasticiser, and multi-scale kenaf fibre as reinforcing filler. The microstructure and the physical, tensile, and water barrier properties of corn starch reinforced with kenaf fibre were characterised and investigated. The biocomposite films were developed via the solution casting technique using 10 g of CS with 0 to 8% kenaf fibre as filler treated with 30% (*w*/*w*, starch basis) of sorbitol. The increased amount of kenaf fibre introduced contributed to improvements in film thickness, weight, and density. Conversely, slight reductions in the biocomposite films’ moisture content, water absorption, and solubility rating were 9.86–5.88%, 163.13–114.68%, and 38.98–25.17%, respectively. An X-ray diffraction (XRD) test revealed that the films were amorphous and that there was no effect on the crystallinity structure of films with kenaf fibre reinforcement. Fourier transform infrared (FT-IR) and rheological analysis indicated that kenaf fibre could weaken the molecular interaction of the film matrix. Field emission scanning electron microscope (FESEM) revealed the arrangement and uniform distribution of kenaf fibre at 0.2–0.8%. The incorporation of kenaf increased the tensile strength, Young’s modulus, and elongation at break until (6% wt) of fibre. With the kenaf fibre incorporation, the optimal tensile strength, Young’s modulus, and elongation at break of the films reached 17.74 MPa, 1324.74 MPa, and 48.79%, respectively. Overall, the introduction of kenaf fibre as filler enhanced the physical and mechanical properties of CS films.

## 1. Introduction

The rapid spread of the pandemic [1] has triggered a sharp increase in the plastics demand [2]. It is shown that the extensive usage of plastic-based products around the world is tied to the large-scale disruptions of the upstream supply chain and huge consequences to the downstream waste disposal system [3,4,5,6,7]. Polyethylene packaging is responsible for over a third of all plastic polymers production [8] and accounts for over 42% of the total plastic demand in the USA and 40% of the plastic demand in Europe [9].

In keeping with the United Nations Environment Programme’s (UNEP) recommendations [10], the vast majority of plastic packaging consists of single-use plastics, including shopping bags, containers, and bottles. Plastic goods are typically disposed of within a year after being made because they are made for disposal [10]. The demand curve for different plastic items should move in lockstep with the pandemic curve for plastic-based PPE, e.g., surgical gloves and face masks for healthcare workers, disposable plastic components for life support equipment, respirators, and general plastic materials [11,12].

In an attempt to resolve the ongoing environmental crisis caused by long-term biodegradable plastics, biopolymers have been considered as potential substitutes for traditional plastics [13,14,15,16]. It is known that starch is the most common polymer, which is widely used as a matrix for composite materials. In addition to being widely available, it is renewable, inexpensive, and biodegradable. However, thermoplastic starch (TPS) exhibited poor water barrier and mechanical properties [17,18,19]. These might be due to the greater hydrophilicity and affinity to water, which hindered their applications [20]. Previously, most of the studies focused on the variety of additives and biopolymer film-forming thermoplastic starch mixtures. For example, Wang et al. [21] investigated thermoplastic starch (TPS) film with corn starch and urea plasticiser by varying the quantities of glycerol, sorbitol, and poly(vinyl alcohol). Moreover, Isotton et al. [22] and Ibrahim et al. [23] investigated the qualities of corn starch-based films and discovered that different plasticisers had different impact properties. Hazrol et al. [24] found that the incorporation of 30% of sorbitol within corn starch resulted in an improvement in mechanical properties with 13.62 MPa and 495.97 MPa for tensile stress and tensile modulus, respectively. In addition, many applications have begun to utilise lignocellulosic fibres with polymers due to their particular environmental advantages as well as improved the mechanical properties of biocomposite [25,26,27,28]. It should also be noted that the link between lignocellulosic fibre and the starch matrix is also one of the important criteria. These attributes are vital in promoting both mechanical strength and hydrophilic sensitivity [29]. Furthermore, including these fillers in composite materials to increase their reinforcing capacity is a wise move.

Kenaf has now become a viable fibre source that can be made from the entire plant, not only the leaves [30]. The third greatest economically important fibre crop is kenaf (*Hibiscus cannabinus* L.; *Malvaceae*) [31]. Mohanty [32] used agriculture residues as reinforcement. Polymer/polymeric components are often utilised in combination with plant-based fibres to boost mechanical qualities [33]. Aziz and Ansell [34] noticed that various types of natural resources have quick development from prior years, including kenaf plants. Kenaf fibre was developed by Akil [35], who successfully developed kenaf fibre, which offers rapid growth, low cost, and diverse applications in many climates. The kenaf fibres might potentially be used as reinforcement composites to replace synthetic fibres and additionally reduce waste. Thus, these assist in protecting the environment.

This study aims to design and implement the use of kenaf fibre as a filler reinforcement in corn starch-based biocomposites. It is part of an effort to see if plant residues from kenaf crops may be used to help lessen the amount of waste, to assist meet the community’s demand for agricultural and polymer waste disposal, and to benefit the economy by diverting residue into money [36]. The corn starch biocomposite films reinforced with kenaf fibre add to the usefulness of waste items and boost the environmental sustainability of starch-based biocomposites.

## 2. Methodology

### 2.1. Materials

The commercial corn starch was purchased from Thye Huat Chan Sdn. Bhd. The sorbitol plasticisers were provided by Evergreen Engineering & Resources Sdn. Bhd. The starch was in the form of powder basis and graded using a sieve machine (A060-01, Matest, Treviolo, Italy) to a size of 0.25 mm. Kenaf fibre was received from Lembaga Kenaf dan Tembakau Negara and graded using rotor mill machine (Pulverisette 19, Fritsch, Idar-Oberstein, Germany) to 0.25 mm in size.

### 2.2. Preparation of Biocomposite Films

**Figure 1:**
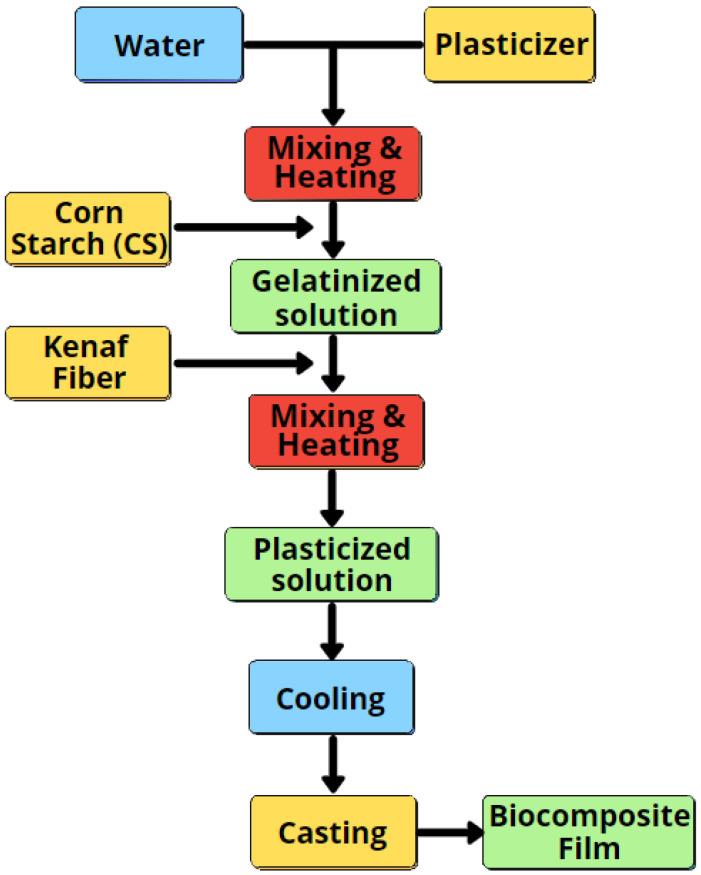
Preparation flow of corn starch biocomposite film reinforced with kenaf fibre.

The corn starch-based biocomposite films were prepared via standard solution casting procedures, as presented in Figure 1. The 30% sorbitol (*w*/*w*, starch basis) plasticiser was used and mixed into a beaker with 180 mL of distilled water. To produce a homogeneous solution, the beaker was then placed into a water bath at 85 ± 5 °C for about 20 min. Following that, 10 g of pure corn starch was added to the solution and heated for another 20 min. After that, kenaf fibre was introduced into the solution at 2, 4, 6, and 8% wt of corn starch and stirred for 20 min. The hot slurry was left to cool prior to the casting process on a thermal plate. The weights of the casting dishes were then fixed at 45 g to produce films with uniform thickness. The blend was oven-dried at 65 °C for 15 h in an air circulation oven. The biocomposite films were coded as CS/K2%, CS/K4%, CS/K6%, and CS/K8%, whereas the control films were coded as CS/K0%.

### 2.3. Film Weight

Film weight was measured using analytical balance ME104E with a readability of 0.1 mg. Five replicates for each film were calculated for each sample. The average values of the measurements were then used as the result.

### 2.4. Film Thickness

Film thickness was measured using a 0.001 mm-sensitive digital micrometre (Mitutoyo Co., Kawasaki, Japan) for each film sample in five different film areas. The thickness values were taken as the average measurement values.

### 2.5. Film Density

The films’ density was measured using a densimeter (Mettler-Toledo (M) Sdn. Bhd., Shah Alam, Malaysia). The initial dry weight of each film was recorded. Xylene was used as the dipping solvent used to replace distilled water to avoid water uptake by the hydrophilic film samples. In addition, the dipping solvent must have a density less than the film to prevent the films from floating on the solvent’s surface, making xylene an ideal option. After the immersion, the film samples underwent drying in desiccators for seven days using SiO2 as the drying agent. The immersed films were reweighed and recorded as (m). The test was performed in three replicates. The amount of liquid displaced by the film was denoted as volume (V). Equation (1) was used for the density calculation (ρ).

ρ = m/v(1)

### 2.6. Film Moisture Content

The initial film samples weight (Wi) was measured using a digital balance. Then, they were oven-dried at a temperature of 105 °C for 24 h, and then reweighed (Wf). The test was conducted in triplicate, and the moisture content was calculated as the mean value using Equation (2).

Moisture content = (W_i_ − W_f_)/W_i_ × 100 (2)

### 2.7. Water Absorption (WA)

A 15 mm^2^ film sample was oven-dried at 105 °C for 3 h, cooled, and instantly weighed (Mi). The sample was then immersed in 100 mL of distilled water at room temperature. After a particular immersion time, the sample was taken out of the water, wiped with a smooth cloth, and reweighed (Mf). Three test duplicates were carried out, and the mass changes between the initial and the immersed films were used to determine the WA using Equation (3).

WA (%) = ((M_f_ − M_i_)/M_i_) × 100 (3)

### 2.8. Water Solubility (WS)

This test was carried out in compliance with the procedures outlined in [37]. The sample was dehydrated and weighed after being left in the dehydrator for 24 h at 105 °C (Wi). The water sample was added to a beaker containing concentrated water that was stirred continuously at room temperature for 12 h. A constant weight (Wf) was obtained by drying the insoluble sample at 105 °C until the sample had no detectable residual mass. Equation (4) shows the WS (percentage) of the sample has been measured as follows.

WS (%) = ((W_i_ − W_f_)/W_i_) × 100 (4)

### 2.9. Fourier Transform Infrared Spectroscopy (FTIR)

Infrared spectrometer models (Bruker vector 22, Lancashire, UK) were used to detect the presence of functional groups. The spectrum observed during the test had a 4000 to 650 cm^−1^ range and a spectral resolution of 4 cm^−1^. A layer of potassium bromide was then added to the samples, and the resulting slurry was squeezed into thin, clear sheets that were then analysed.

### 2.10. X-ray Diffraction (XRD)

A Shimadzu LabX XRD 6000 (Kyoto, Japan) was used for the XRD test. The new software suite X’Pert HighScore Plus, an integrated software platform that integrates both measurement and analysis functions, was used for the measurement. A scattering angle speed of 1° (θ) min^−1^ within angular values from 5° to 40° (2θ) was used as the operating condition. The tube voltage and current were set at 40 kV and 35 mA, respectively. The outcomes from the XRD test included relative crystallinity (X_c_), crystalline area (I_c_), and amorphous area (Io). Equation (5) presents relative crystallinity as a ratio between crystalline and amorphous space.

X_c_ (%) = ((I_c_ − I_o_)/I_c_) × 100 (5)

### 2.11. Tensile Properties of Films

A universal tensile machine (5kN INSTRON, INSTRON, Norwood, MA, USA) characterised the mechanical characteristics of the specimens. The test was performed following the ASTM method [38]. A film strip (70 mm × 10 mm) was fixed to the machine’s clamps and pulled at 2 mm/min crosshead speed and at an effective grip distance of 30 mm. The tensile machine was automated by a computing software, Bluehill 3, that used a mean calculation from five replicates for each specimen to obtain the findings of elastic modulus, tensile power, and elongation at the breakpoint.

### 2.12. Field Emission Scanning Electron Microscopy (FESEM)

The samples’ surface morphology analysis was conducted using a high-resolution field emission scan electron microscope (FEI NOVA NanoSEM 230, FEI, Hillsboro, OR, USA). Each sample was first made conductive to currents by coating the sample with a thin gold layer (1.5–3.0 nm) using an argon plasma metalliser (sputter coater K575X, Crawley, UK) prior to the examination to prevent charging. The scans were conducted at 3 kV accelerating voltage to examine the micro- and nanostructure surfaces of the longitudinal cross-sections of the kenaf fibre and corn starch [39].

## 3. Results and Discussion

### 3.1. Physical Appearance of Corn Starch/Kenaf (CS/K) Biocomposite Films

**Figure 2:**
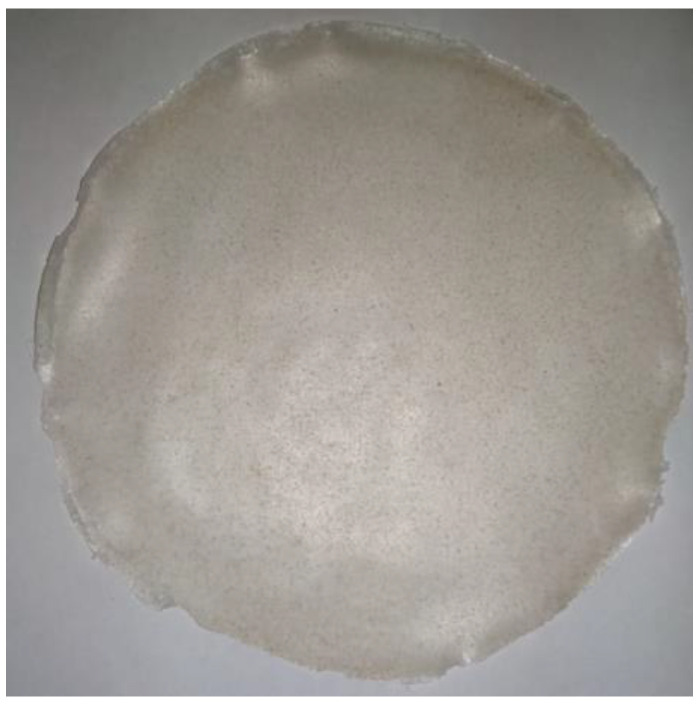
Corn starch biocomposite film reinforced with kenaf fibre.

Figure 2 presents the photographic images of the corn starch biocomposite films reinforced with kenaf fibre, while Table 1 lists their visual appearances from control film to CS/K biocomposite films. The control corn starch films prepared using a 30% sorbitol plasticiser was rigid, crystal clear, not brittle nor fragile, non-sticky, peelable, and flexible. This finding could be the result of weak inter-/intramolecular hydrogen CS bondings that resulted in rigid, not brittle, and flexible films, giving the macromolecular chains more movement. These findings were aligned with those from Suppakul et al. [40]; Sanyang et al. [41]; and Norizan et al. [42] on the investigation of cassava, potato, and sugar palm starches, respectively. In addition, the morphology of all multi-scale corn starch biocomposite films reinforced with kenaf fibre is summarised in Table 1.

### 3.2. Physical Properties of CS/Kenaf Biocomposite Film

In Table 2, the thickness of CS biocomposite films reinforced with kenaf fibre demonstrated a constant thickness up to 6% of kenaf fibre loading. A slight increase in thickness value was also observed at 8% of kenaf fibre loading. This finding could be explained by the role of fibres in the reconstruction and restructuring of intermolecular chain networks, resulting in enhanced film thickness. This finding was similar to that investigated by numerous literatures [43,44,45,46,47].

Furthermore, the CS/K2% and CS/K4% biocomposite films showed lower weight at 0.07 mg compared to CS/K6% and CS/K8% biocomposite films weighing 0.8 mg. The increasing thickness and weight of films with a high amount of fibre films might be due to the high molar mass of kenaf fibre. Sanyang et al. [44] also stated that an increased amount of fibre in films yielded thicker films.

The effect of fibre loading on the density of CS/K films is presented in Table 2. Upon close observation, it appears that increasing the kenaf fibre loading from 2 to 8% has no effect on the film density. Intermolecular interactions between the fibre and the polymer matrix might be responsible for this observed finding. The samples behaved similarly with those previously loaded with cassava starch film and then filled with cassava bagasse [48], the production of sugar palm fibre and starch [49], and the reinforcement of sugar palm starch composites by seaweed fibre [50]. It is known that biocomposites have lower densities and are suitable for lightweight and simple applications [51].

It was discovered that the CS film is hydrophilic in nature [24]. Although some kenaf fibre was included in the CS biocomposite films, the incorporation of kenaf fibre to some extent helped to lower the amount of water retained by the CS biocomposite films, as seen in Table 2. A possible correlation could be that kenaf fibre’s moisture content was lower, which would explain the somewhat reduced moisture content of the composite films. Consistent results were noted by Lopez et al. [52], who reinforced wheat starch with leaf wood fibre [23], for reinforcing corn starch with corn husk fibre, and similar to those [53] using fibrous residues from *Pachyrhizus ahipa* plant to corn starch. The mechanical behaviour of the samples was evaluated using a universal tensile machine (5kN INSTRON). The test was performed in accordance with the ASTM method [38]. A film strip (70 mm × 10 mm) was clamped to tensile machine clamps and pulled at 2 mm/min crosshead speed and at an effective grip distance of 30 mm. The machine was linked and automated by a computational software, Bluehill 3, that assessed the findings of tensile power, elastic modulus, and elongation at the breakpoint using a mean calculation of five replicates for each specimen.

### 3.3. Water Solubility (WS)

Both Table 2 and the composite film’s water solubility illustrate the influence of steady stirring at 500 rpm for water immersion on the composite films. Increasing the level of CS/K (2% and 4%) resulted in higher water solubility, but further increasing the fibre level caused a decrease in water solubility. This might happen because of the fibre’s role as a network, holding composite films together and lowering their solubility. This was in agreement with the findings of Edhirej et al. [54], who investigated the cassava starch/peel biocomposites, and [23] who studied corn starch-based using multi-scale corn husk fibre. The two researchers asserted that low fibre loading films were more soluble than high fibre loading films.

### 3.4. Water Absorption (WA)

**Figure 3:**
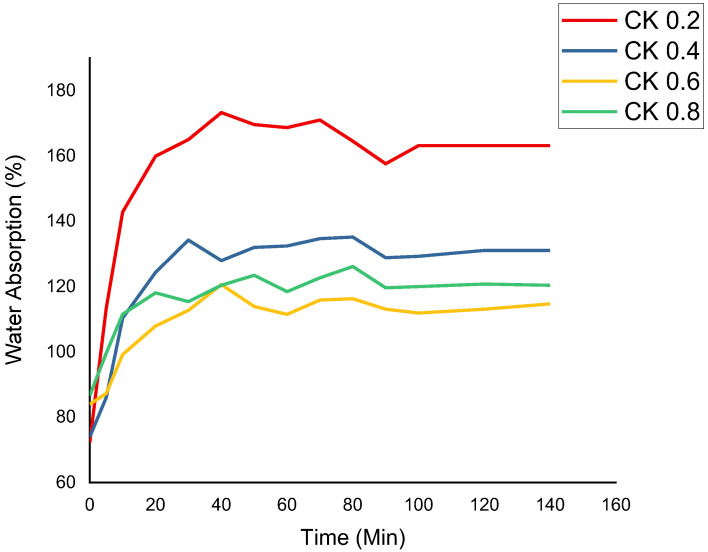
Water absorption in CS biocomposite films reinforced with kenaf fibre.

Figure 3 displays the weight gain percentage based on water intake for the CS biocomposite films reinforced with kenaf fibre. This is because after 140 min, the films began to degrade. However, water intake was nearly uniform across CS/K 2%, CS/K 4%, and CS/K 8% with higher fibre loading. In contrast to films reinforced with CS/K6% that showed less tendency towards water intake, the films that utilised CS/K6% demonstrated less inclination towards water consumption. It was suggested that films containing 2, 4, and 8% of kenaf fibre absorbed more water than films containing 6% of kenaf fibre. It was found that, with regards to behaviour, [55] used chitin as filler into thermoplastic corn starch-based composite, and [23] used corn husk fibre as filler into corn starch-based composites. Both agreed that the water absorption rate of the biocomposites, which were formed from thermoplastic and corn husk fibre matrix, was reliant on the filler percentage in the polymer and corn husk fibre matrix. Resistance to water absorption was higher in biocomposites filled with kenaf fibre than thermoplastic or corn husk fibre. Besides that, Ilyas, Sapuan, Atiqah, et al. [56] found that sugar palm fibre’s high sugar content would lead to better water absorption in composite films made from sugar palm starch. Because of the hygroscopic nature of sugar palm fibre, it was postulated that the phenomenon occurred. Even though the researchers believe this characteristic is associated with the hydrophilic properties of CS and kenaf, it might just be that the finding was influenced by the use of hydrophilic CS and kenaf. Fibre inclusion has the effect of enhancing the starch’s porosity, which enables water to permeate more readily [57,58].

### 3.5. Fourier Transform Infrared Spectroscopy (FTIR)

**Figure 4:**
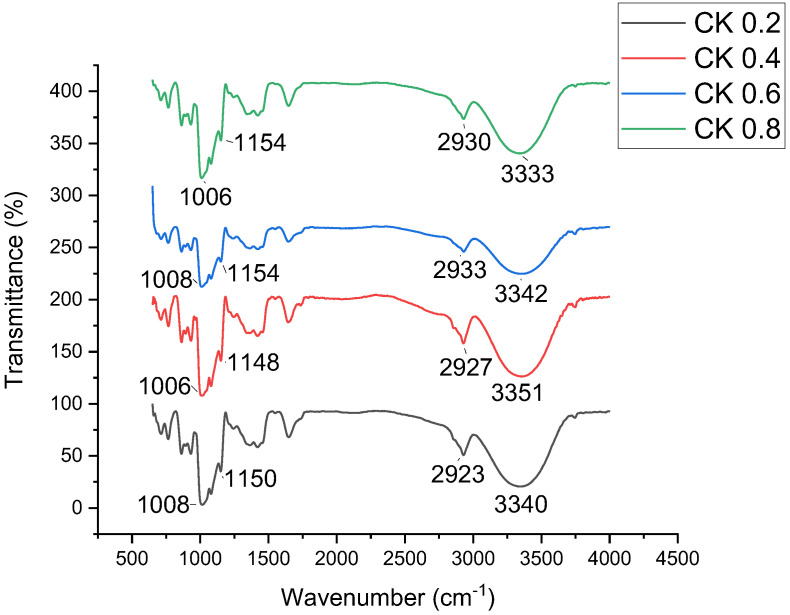
FTIR curves of CS/K films with multi-scale kenaf fibre loading.

Figure 4 displays the FTIR spectrum of CK samples. The FTIR was used to investigate the differences in starch compositional structure and to identify possible interactions between plasticiser and fibre [55,59,60]. The FTIR spectrum curve was divided into four main parts: wavenumbers above 3000 cm^−1^, from 2800 to 3000 cm^−1^, from 800 to 1500 cm^−1^, and up to 800 cm^−1^ and below. A strong band appeared at approximately 3333 to 3351 cm^−1^ in most of the CS/K samples. The CS/K6% biocomposite film showed peaks at 1008, 1154, 2933 cm^−1^, and the maximum peak around 3342 cm^−1^ was ascribed with the O-H groups’ stretching. A polymer chain of kenaf-reinforced CS was identified at the band of 2933 cm^−1^, which was associated with C-H stretching. The peak at 1154 cm^−1^ was due to the bending of the absorbed water O-H and C-O bonding. Meanwhile, the peak at 1008 cm^−1^ demonstrated the C-H molecules’ initiated vibration, resulting in H atoms separated from C. This result is consistent with the treated fibre structure, where the lignin content decreased in bleached kenaf fibre [59].

### 3.6. X-ray Diffraction (XRD)

**Figure 5:**
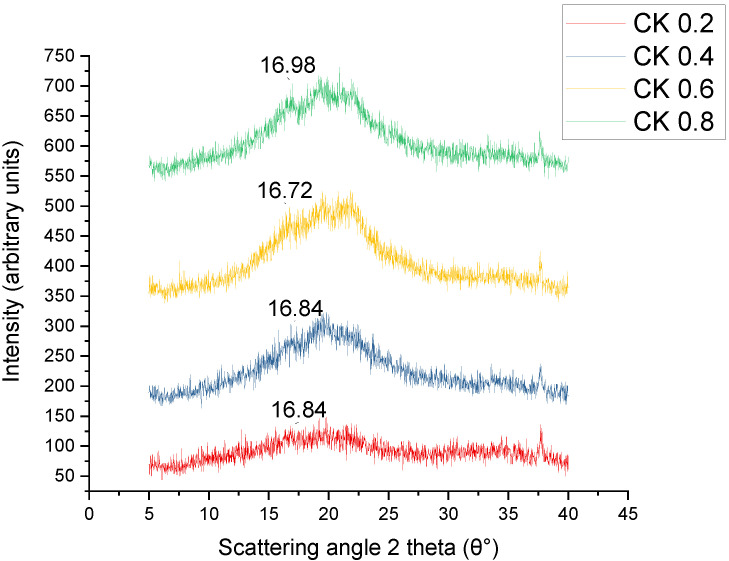
XRD of CS/K biocomposite films.

The XRD analysis was conducted to characterise the crystallinity of biocomposite films, as demonstrated in Figure 5. The CS/K samples demonstrated clear peaks at 16.84°, 16.72°, and 16.98°, which were aligned with the diffraction peaks characteristics of starch and fibre. These findings were consistent with those reported by Paraginski et al. [60], indicating that the CS/K sample commonly maintained the fundamental amorphous structure of starch and fibre [7,43,47,61]. However, a slight difference in the diffraction patterns was observed. The CS films revealed crystallinities of 10 to 20° reflectance, similar to the B-type diffraction pattern [62]. Zhong and Li [63] noted similar findings where they proposed the development of double-helical crystalline B-type with peaks at 2θ to 17°.

The initial addition of kenaf fibre, especially at 2%, showed a similar diffraction profile to the control and caused a slight increase in relative crystallinity. As expected, the further addition of fibre improved the intensity and crystallinity index of the biocomposites, as shown in Table 3. According to Ma et al. [61] and Edhirej et al. [54], the increase in the crystallinity of fibre reinforcement composite could be attributed to the development of phase separation between the fibre and starch, which indicates that the structure of fibre composite has become more organised and less amorphous. One theory indicated that the thermoplastic cassava starch/peel fibre composites work in a similar manner [54] with thermoplastic corn starch/bacterial cellulose composites [64] and corn starch/corn husk fibre composites [65]. The results of X-ray diffractometry testing determined by applying the two methods mentioned earlier confirmed that kenaf contributed to a lower relative crystallinity in the CS-based composites. Table 3 shows that as the fibre content increased, the crystallinity increased. The crystallinity degree of the control film was 39.86%; the gradual increase in fibre resulted in an increase in the crystallinity degree, which reached 48.51% with 8% husk content.

### 3.7. Tensile Properties

**Figure 6:**
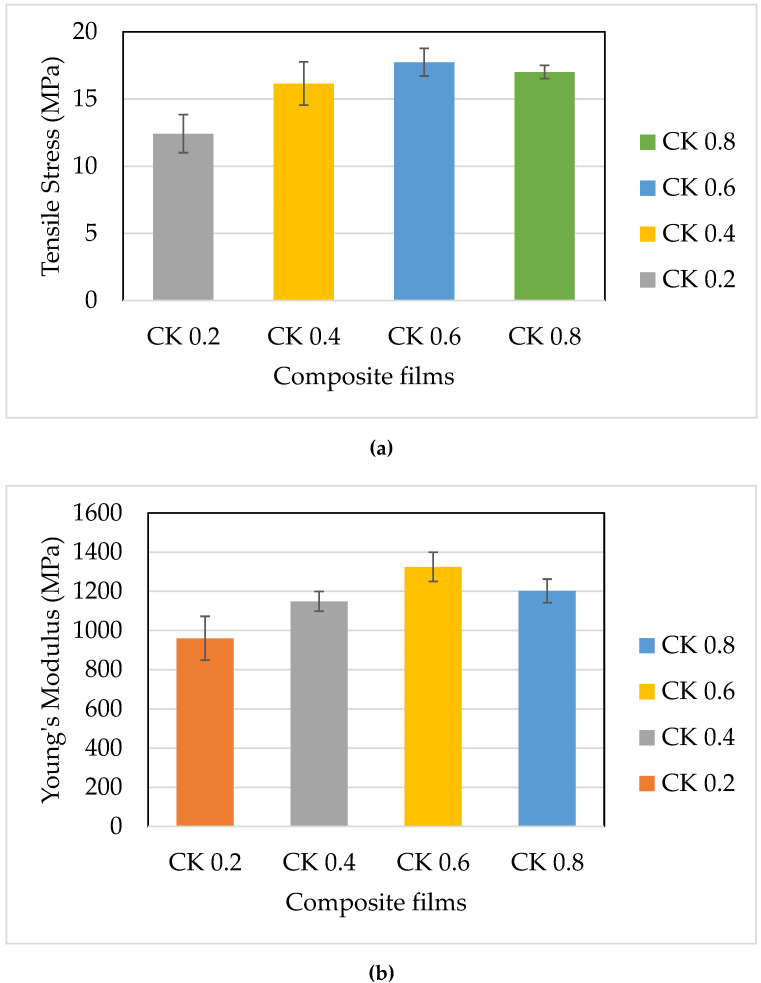

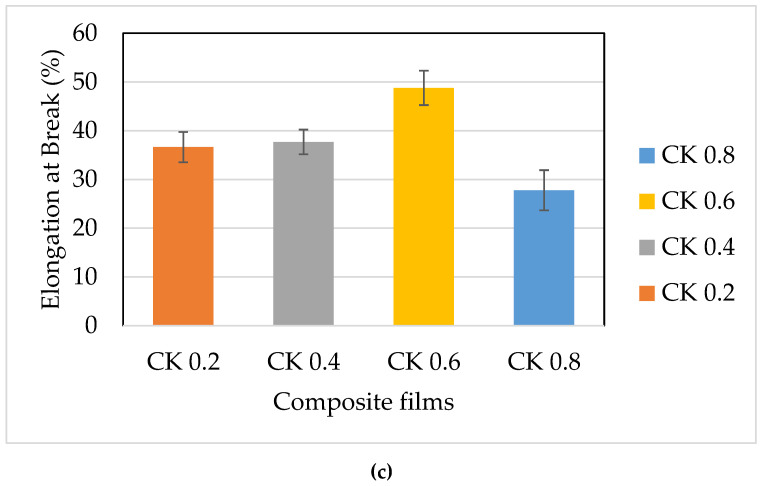
Tensile properties of corn starch biocomposite films reinforced with kenaf fibre. (**a**) Tensile strength, (**b**) tensile modulus, and (**c**) elongation at break.

The tensile performance of CS-based films is demonstrated in Figure 6. Here, the tensile strength (TS), tensile modulus (E), and elongation at break (EB) were investigated. The results demonstrated that the tensile strength of the tested films raised proportionally as the fibre loading was raised, which was greatly aligned with the previous hypothesis [24,66]. The CS/K6% biocomposite film recorded the maximum tensile strength (17.74 MPa), higher than CS/K8% (17.01 MPa). This is related to the creation of hydrogen bonds between starch and plasticiser molecules, which were dominated by lower plasticiser content and decreased as plasticiser content raised [23,67,68,69,70]. The CS/K biocomposite films possessed tensile strength commonly higher than the previously recorded values for glycerol-plasticated corn films, corn starch with stearic acid and glycerol, and corn starch with xylitol and glycerol [71]. In contrast, the tensile strengths for CS films were higher compared to sweet potato starch added with thymol [64].

Figure 6b shows the effect of kenaf fibre loading on the modulus of the CS-plasticised film module. It can be observed that similar behaviour was demonstrated corresponding to their tensile strength. Increasing the kenaf loading from 2 to 6% resulted in an increase in film stiffness from 959.8 to 1324.74 MPa, and it decreased back to 1201.88 MPa for CS/K8%. The fibre function can explain this behaviour by modifying the structure of the starch network. Incorporating fibre into starch chains as filler facilitated the hydrogen bonds formed between molecules and strengthened the solid intra-molecular attraction within the starch matrix. Young’s CS-plasticised film modulus was reduced and less rigid [23,44,54,71,72,73].

Similarly, it was shown that adding fibre to CS-based composites led to the same increase in their modulus and tensile strength, of 36.63 to 48.79% for CS/K2% to CS/K6%, and a decrease to 27.79% for CS/K8%. This statistic, known as deformation capacity, was used to quantify the composite films’ ability to deform and stretch from their original length up to their breaking point [37]. This apparent decrease in the film’s elongation as the fibre loading increases was caused by the increased intermolecular linkages formed between cellulose and starch molecules. Manufactured products made of starch require rigidification by starch network rebuilding. As a result, flexibility and elasticity are lost, and products have rigidity [60].

### 3.8. Field Emission Scanning Electron Microscopy (FESEM)

**Figure 7:**
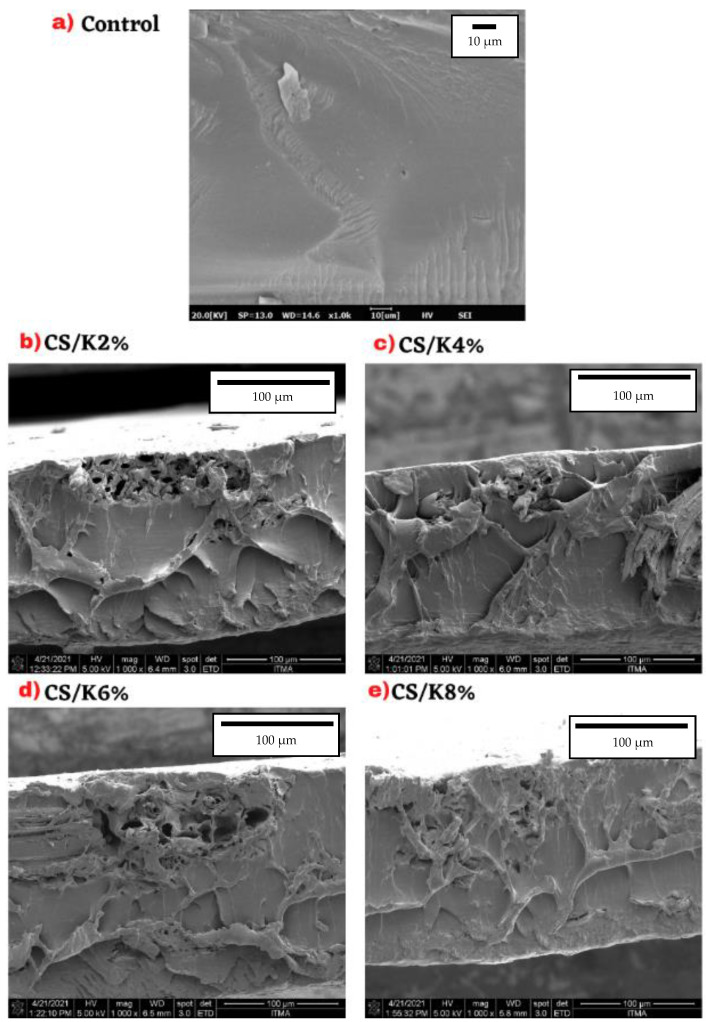
Field emission scanning electron micrograph of CS/K biocomposite films with various kenaf fibre loading and concentration values. (**a**) Control film, (**b**) CS/K2% biocomposite film, (**c**) CS/K4% biocomposite film, (**d**) CS/K6% biocomposite film, and (**e**) CS/K8% biocomposite film.

When it comes to fibre strength, often-referenced aspects are fibre orientation and arrangement. The morphology of CS, CS/K2%, CS/K4%, CS/K6%, and CS/K8% was analysed using FESEM. The FESEM images of the fractured cross-sectional surfaces of CS and CS/K film samples at 1000× magnification are shown in Figure 7. The microstructures of the film surfaces were examined to compare the surface morphology of CS and multi-scale kenaf fibre loadings. Generally, the control film had consistent homogeneous surfaces and a clear plasticiser cover without any visible fibres [74]. This was because of the plasticiser that functioned as a strong interaction bonds builder within the starch matrix, since the plasticiser and matrix were both carbohydrates of the same polarity [75]. SEM of native CS presented a relatively smooth and uniform surface, reflecting the CS morphological structure [43,72]. This smooth surface was a result of the perfect and homogeneous stirring during the film preparation.

The addition of the kenaf fibre K2%, K4%, K6%, and K8% in the CS film demonstrated such disturbances to film cross-sectional surfaces. Studies found that when the kenaf fibre content was above 8%, there were microcracks and longitudinal holes in the starch matrix. For the purpose of analysing the overall structure of a film, the authors look for matrix uniformity [73,76]. It can be seen that the increasing amounts of kenaf fibre is believed to transmit stress from the fibre to the substrate, hence creating improved mechanical qualities [50,77]. The addition of fibre to corn starch gives the film a better structure and quality [78]. The similarities in hydrophilicity of the matrix and reinforcement may explain the constant dispersion and coherency in the fibre interactions observed [79]. The material, thermoplastic cassava starch/cassava bagasse biocomposites, had a comparable surface fracture discovered on it [48].

## 4. Conclusions

In this work, the tensile strength of the films was determined to suggest the best possibilities of eco-friendly composites for various domestic applications. From the results, it can be noticed that composites made of 6% of kenaf fibre demonstrated better tensile properties than all other fibre loadings. It was also observed that the distribution of fibre and corn starch revealed well-structured fillers and matrices. Conversely, slight reductions in the biocomposite films’ moisture content, water absorption, and solubility rating of 9.86 to 5.88%, 163.13 to 114.68%, and 38.98 to 25.17%, respectively, were observed. X-ray diffraction (XRD) showed that the films were amorphous, and kenaf had no effect on the crystallinity structure of the films. The incorporation of kenaf increased the tensile strength, Young’s modulus, and elongation at break up to (6% wt) of kenaf fibre. The best composition is CS/K6%. With the kenaf fibre incorporated, the optimal tensile strength, Young’s modulus, and elongation at break of the films reached 17.74 MPa, 1324.74 MPa, and 48.79%, respectively. Therefore, it is suggested that introducing kenaf fibre as filler could solve the fragility and brittleness of CS films, and thus have huge potential to replace current petroleum-based plastic.

## Figures and Tables

**Table 1:** Physical appearance of biocomposite films.

Label	Plasticiser	Kenaf Fibre Loading (%)	Appearance of Films
Control	Sorbitol	0	Crystal clear, rigid, not brittle nor fragile, non-sticky, flexible, peelable.
CS/K2%	Sorbitol	2	Brown colour, large fibre gap present, non-sticky, rigid, not brittle nor fragile, peelable, flexible.
CS/K4%	Sorbitol	4	Brown colour, moderate fibre gap present, rigid, not brittle nor fragile, non-sticky, more flexible than CS/K2%, peelable.
CS/K6%	Sorbitol	6	Brown colour, less fibre gap present, non-sticky, rigid, not brittle nor fragile, most flexible among all, peelable.
CS/K8%	Sorbitol	8	Dark brown colour, less fibre gap present, not brittle nor fragile, non-sticky, more flexible than CS/K2% and CS/K4%, easy to peel.

**Table 2:** Physical properties of corn starch films incorporated with various plasticiser types and concentration.

Fibre Loading (%)	Thickness (mm)	Weight (mg)	Density (g/cm^3^)	Moisture Content (%)	Water Solubility (%)
Control	0.16 ± 0.02	0.08 ± 0.02	1.45 ± 0.05	9.25 ± 2	25.17
Kenaf 2%	0.16 ± 0.02	0.07 ± 0.02	1.42 ± 0.05	9.86 ± 2	38.96
Kenaf 4%	0.16 ± 0.02	0.07 ± 0.02	1.45 ± 0.05	7.60 ± 2	37.28
Kenaf 6%	0.16 ± 0.02	0.08 ± 0.02	1.45 ± 0.04	5.99 ± 2	33.67
Kenaf 8%	0.17 ± 0.02	0.08 ± 0.02	1.45 ± 0.03	5.88 ± 2	25.17

**Table 3:** Crystallinity index of CS/K biocomposite films. Physical appearance of biocomposite films.

Film Sample	Crystallinity Index (%)
Control	39.86
CS/K2%	40.33
CS/K4%	43.71
CS/K6%	45.06
CS/K8%	48.51

## Data Availability

Not applicable.
